# Cervical Implant Allergy With Chronic Neck Pain: A Case Report

**DOI:** 10.7759/cureus.28293

**Published:** 2022-08-23

**Authors:** Ryoma Aoyama, Ukei Anazawa, Hiraku Hotta, Itsuo Watanabe, Yuichiro Takahashi, Shogo Matsumoto

**Affiliations:** 1 Orthopaedics, Tokyo Dental College Ichikawa General Hospital, Ichikawa, JPN

**Keywords:** postoperative neck pain, neck pain, allergy, spinal implant, spinal instrumentation

## Abstract

A 57-year-old woman underwent cervical implant surgery for a dislocated cervical spine fracture, and she complained of continuous intractable neck pain after surgery. Eight years later, she developed a plantar skin rash, subsequently diagnosed as a metal allergy, and metal dentures were replaced with ceramic ones. The skin rash, however, persisted for four more years after that and was eventually treated with cervical implant removal. Subsequently, her skin rash and her neck pain improved simultaneously. This synchronous improvement strongly suggested that the neck pain could have been caused by a cervical implant allergy. We discuss a case of posterior cervical implant allergy that presented with neck pain and plantar skin rash years after surgery.

## Introduction

Metal allergy is a well-known complication after instrumentation surgery [1]. In a study by Kitagawa et al. involving 925 patients who underwent allergy patch testing for a clinical history of metal allergies, dental metal allergies were recognized as the major cause of their metal allergies, and symptoms appeared in 392 of the 925 (42.4%) patients [2]. There have also been a few case reports of metal allergies after spinal instrumentation surgeries [3-8]. Furthermore, allergies, though fewer in number, have been reported after titanium alloy implants [1,6-8].

Neck pain after posterior cervical spine surgery is a relatively common complaint of a muscular origin [9], and chronic neck pain occurs in approximately 2% of patients [10]. On the other hand, neck pain could be reduced by spinal bony fusion on the surgical site [11].

We present a case of skin rash diagnosed as a metal allergy that appeared eight years after spinal titanium alloy implant surgery and improved four years later after spinal implant removal. Intractable neck pain that had persisted from the cervical implant surgery also improved after its removal. This simultaneous improvement of symptoms strongly suggests that the residual neck pain was due to an allergy to the implant. This case report highlights infrequent allergic reactions following a spinal instrument surgery.

## Case presentation

A 57-year-old woman fell down the stairs at home and hit her face hard against the wall. A nearby doctor diagnosed a nasal bone fracture and treated it conservatively. She subsequently visited her local orthopedic doctor for the neck pain that persisted and was referred to our orthopedic department on the ninth day after the injury.

**Figure 1:**
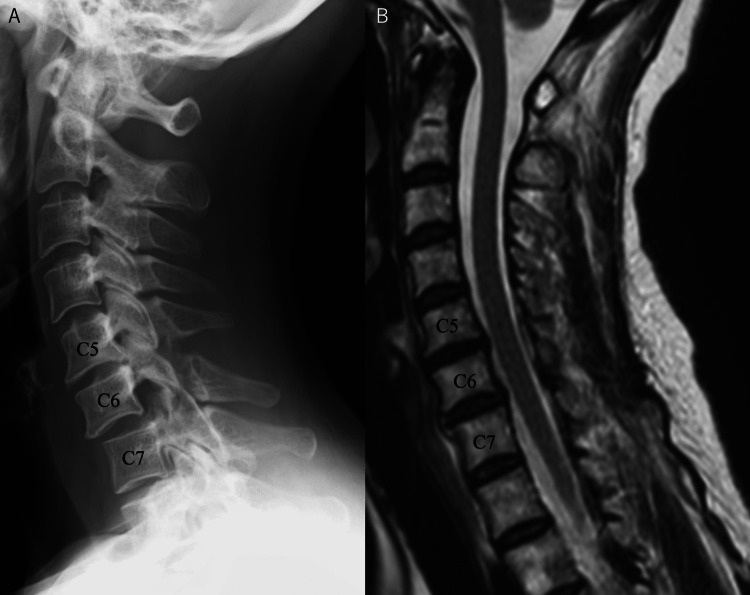
Lateral plain cervical spine X-ray image at the initial examination (A) and T2-weighted MRI sagittal section before the initial surgery (B) A. A plain cervical spine radiographic lateral view showed an anterior C6-C7 subluxation and an increase in the distance of the spinous processes between C5 and C6 B. There was no spinal cord injury, and hemorrhage due to the fracture was not noticeable. A mild disc bulge was seen at C6-C7 greater than at C5-C6 MRI: magnetic resonance imaging

**Figure 2:**
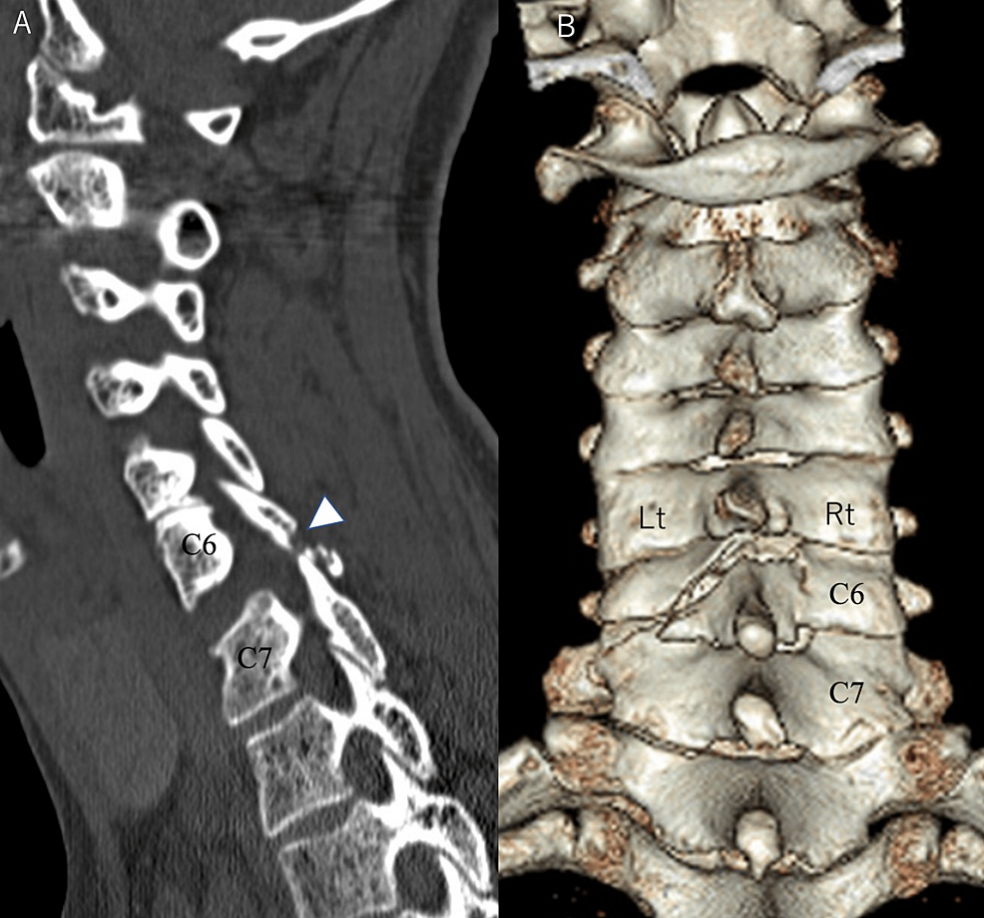
Sagittal CT image before the initial surgery (A) and 3D-CT dorsal view before the initial surgery (B) A. A fracture (arrowhead) of the left C6 inferior articular process was observed, and the C6/C7 facet joint was subluxated B. The C6 lamina showed a lamina fracture on the left side, with the fracture line running from the superior-medial lamina to the caudal lamina-facet joint junction. The right side of the C6 lamina also showed a fracture, but there was only a slight dislocation CT: computed tomography

**Figure 3:**
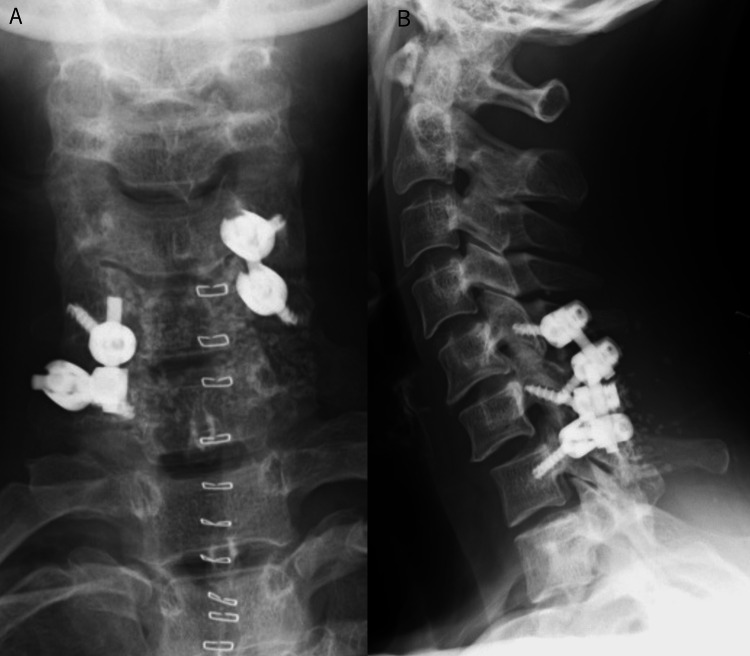
A plain cervical spine anteroposterior X-ray image after cervical spinal implant surgery (A) and a lateral plain cervical spine X-ray image after cervical spinal implant surgery (B) A. The C5-C7 levels underwent fixation with an implant. Bone grafting, including the use of artificial bone, was performed on the dorsal side of the vertebral arch B. A posterolateral fixation of C5-C7 was performed. A small degree of anterior subluxation of C6 onto C7 remained after fixation

There were no neurological deficits at the initial examination; however, her neck pain was evident during body movement. The neck pain did not improve with the use of a neck collar. A plain cervical spine X-ray showed anterior slip of C6 over C7, widened C5-6 interspinous space, perched C6-7 facet, and widened C6-7 spinal foramen (Figure 1A). MRI cervical spine showed small C5-6 and C6-7 cervical disc bulges but no significant spinal canal compromise or cervical cord injury (Figure 1B). CT showed subluxation due to a fracture at the left C6/C7 facet joint (Figure 2). The patient was diagnosed with a displaced fracture of C6, and surgery with a titanium alloy implant was performed in September 2008 (Figure 3).

We initially tried to perform fixation of C5-C7. Since we found it challenging to insert screws at all levels, we performed a right C5-C6 fixation with a lateral mass screw, and a left C6-C7 fixation with a lateral mass screw and pedicle screw to obtain good stability (Figure 3). After surgery, the patient's postoperative neck pain improved, but the intractable pain requiring daily non-steroidal anti-inflammatory drugs (NSAIDs) persisted even after her bone fusion.

Eight years after instrumentation surgery, a skin rash appeared on the sole of her foot. It was diagnosed as palmoplantar pustulosis and treated with phototherapy by a dermatologist, but the rash did not improve. Therefore, the dermatologist considered that the cause of the skin rash was a metal allergy from metal dentures. He performed a patch test and a lymphocyte transformation test. These latter tests confirmed the diagnosis of a metal allergy. All her metal dentures were removed and replaced with ceramic ones. The patient's plantar skin rash did not improve, however, for four more years. The continuing skin rash caused the dermatologist to consider that the cause of this allergy could also be the patient's cervical implant.

**Figure 4:**
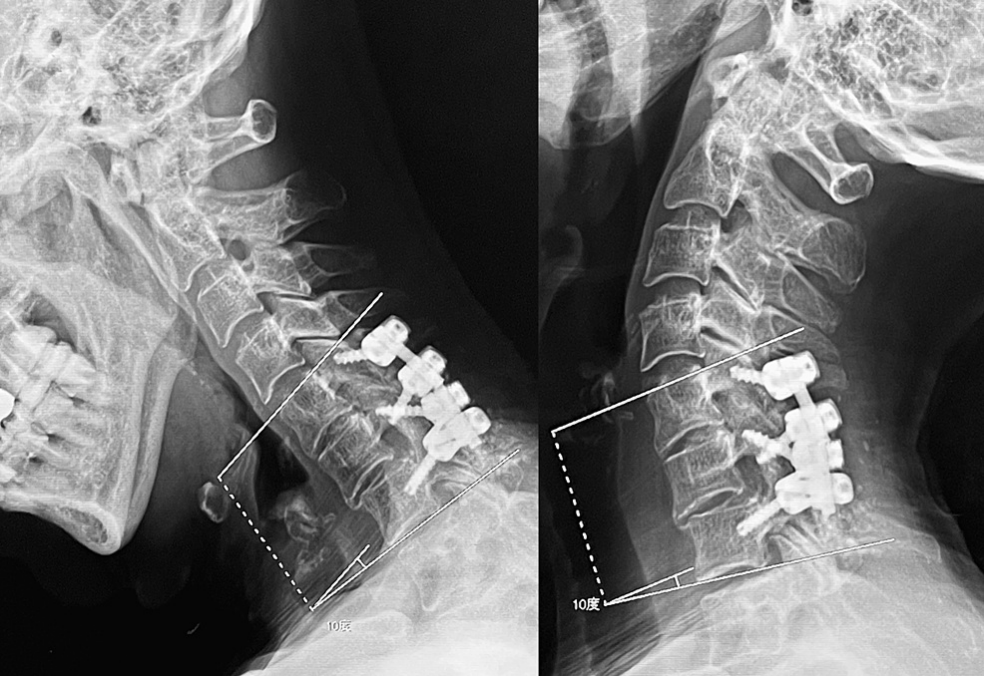
Lateral plain flexion-extension cervical spine X-ray images before removing the implant There was no change in the angle between C5 and C7 when the patient moved her neck from the flexed position on the left to the extended position on the right. The C5-C7 intervertebral space was considered stabilized by a bony fusion

**Figure 5:**
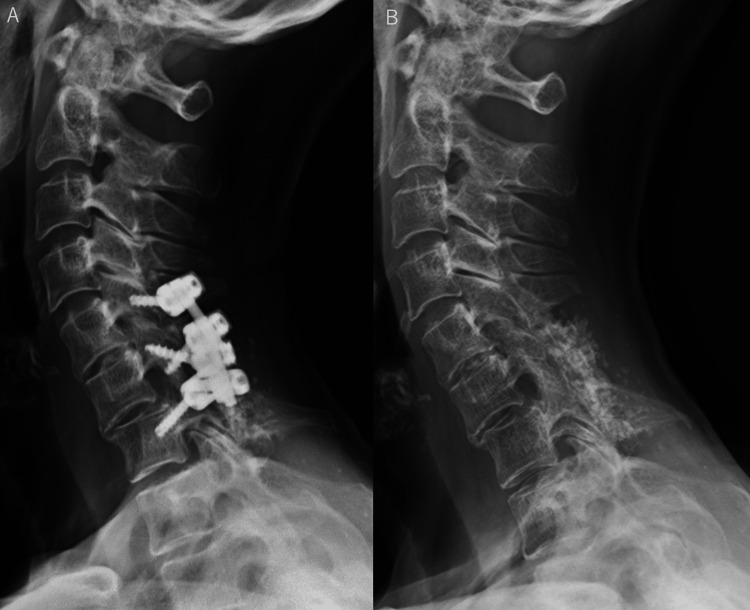
Lateral plain cervical spine X-ray image before removing the implant (A) and lateral plain cervical spine X-ray image at 1 year and 4 months after removing the implant (B) A. Disc space narrowing at C5/C6 and C6/C7 was observed, and this area was considered stable since bony bridging was seen anterior to the C6/C7 disc B. There was no change in cervical alignment after the implant removal, indicating that the bony fusion of C5-C7 was complete

The dynamic plain cervical spine X-rays demonstrated complete stability of the patient's fusion level, allowing implant removal (Figures 4, 5). The patient was thus treated with implant removal 12 years after her original surgery. Complete bony fusion and stability of the fixation site were confirmed intraoperatively.

**Figure 6:**
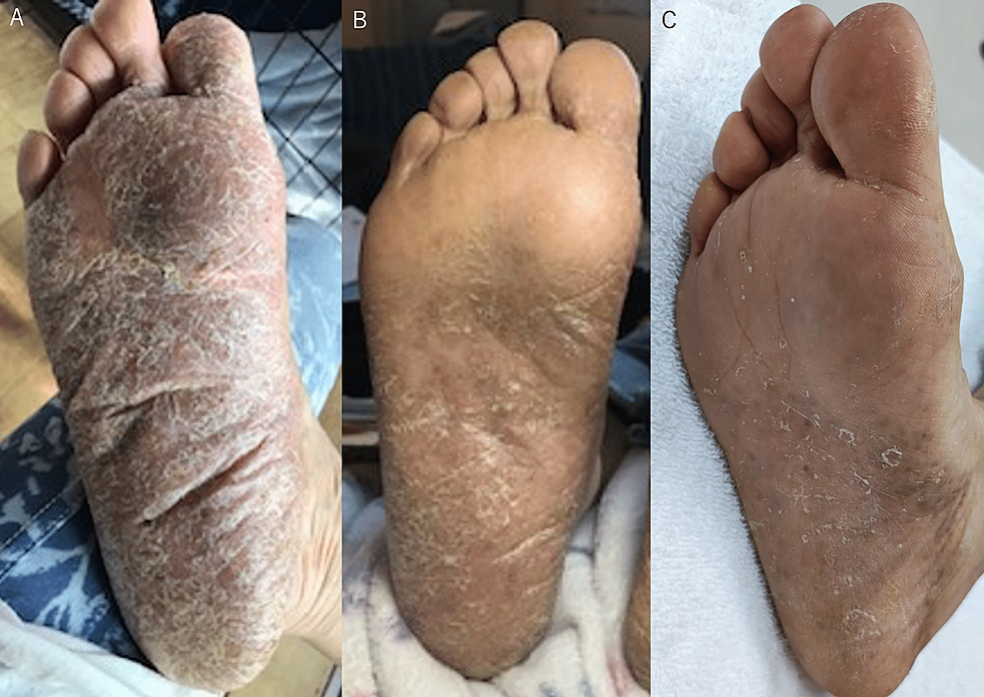
Photograph of the plantar surface of the right foot just before removing the implant (A), photograph of the plantar surface of the right foot 2 months after the implant removal (B), and photograph of the plantar surface of the right foot at 1 year and 7 months after the implant removal (C) A. The rash covered the entire right foot, and the only normal skin area was at the bottom of digits 2-5 B. The amount of skin rash has decreased compared to before the implant removal C. Although the skin rash was still visible, a more normal area was noticeable, i.e., there was a marked decrease in the skin rash

After the implant removal, the skin rash on the sole improved (Figure 6), indicating that the cause of the allergy was the cervical implant. Moreover, her neck pain also remarkably improved at the same time.

## Discussion

A high prevalence of contact allergies to metals has been reported, with various metals as the cause of the allergies in the following proportions: nickel: up to 24.4%; cobalt: 8.8%; chromium: 5.9%; and titanium: 0.2-3% [1]. Hence, the use of titanium implants in recent years has made implant allergies less likely to occur than in the past when cobalt and chromium were usually used. Recently, according to data obtained after implant arthroplasty in approximately 1,000 patients, metal hypersensitivity due to metal implants was as low as less than 1% [1].

Clinically, an international group has proposed the following diagnostic methods for establishing a metal hypersensitivity: confirmation of eruption overlying the implant, positive confirmation of the allergy to a metal component by a patch test, complete clearing of symptoms after the implant removal, and chronic dermatitis that begins weeks to months after implant use [12]. An implant allergy is diagnosed by a patch test once a skin rash is confirmed, and implant removal is essential to both the treatment and definitive diagnosis.

In a study by Mody et al., biopsy specimens of surrounding tissue around the spinal implants of 36 patients undergoing lumbar spinal hardware removal for pseudoarthrosis, prominent hardware, and fusion extension showed soft tissue reactions. Black amorphous metal deposits were seen in nine specimens, of which seven showed an inflammatory foreign-body reaction [13]. In another study by Kasai et al. involving 46 patients with titanium alloy spinal implants, elevated metal concentrations in blood or hair were observed in about one-third of patients, with an average duration of 5.1 years after spinal implant placement. It was suggested that some of the metal from the hardware had dissolved and might cause symptoms in remote organs throughout the body [14].

Immunologically, implant allergy results from autoimmunity/inflammatory reactions and type 4 delayed hypersensitivity reactions [15-17]. The triggering of these autoimmune reactions, hypersensitivity, or inflammatory reactions are attributed to metal-bound proteins recognized by the immune system or by free radical formations produced by metal binding to sulfur in mitochondria, enzymes, and cell proteins [15-17].

Post-spinal-implant metal allergy is a rare entity despite the high frequency of spinal implant reactions. Very few case reports of clinical spinal implant allergy have been published [3-8]. Chronic pain at the surgical site was reported in one case in the cervical region, and three thoracolumbar cases have also been reported. The pain improved in all cases after the implant removal.

Chronic neck pain after a cervical implant is relatively common [10], and pain reduction by implant removal has been reported [18]. The typical symptom of implant allergy is also chronic pain that appears after a low-pain period after surgery [19]. However, in our patient, it was difficult to distinguish between chronic neck pain and symptoms of allergy seen in some cervical implant cases. Some chronic pain after spinal implant surgery could be caused by instability due to a poor bony fusion at the operative site [11] and the patient in this scenario will show significant pain improvement after implant removal [20].

In our case, although the general severe neck pain immediately improved after surgery, chronic neck pain requiring medication continued even after the bony cervical fusion had occurred, and it was considered a complication of surgery. However, a skin rash on the sole appeared eight years after surgery, which revealed the presence of a metal allergy in this patient. This case demonstrated significant pain improvement immediately after implant removal surgery even without the presence of spinal instability due to poor bony fusion. This significant pain relief suggested that this relief was due not only to implant removal itself but also to an improvement in implant allergic symptoms.

## Conclusions

We reported a rare case of a metal allergy after cervical implant surgery with persistent neck pain. Removal of the implant not only relieved the skin rash but also relieved the chronic neck pain that had persisted since the first implant surgery. This report suggests that one should consider metal allergy when a patient complains of refractory chronic neck pain after implant surgery. In such cases, a patch test and a lymphocyte transformation test should be considered.
